# Long noncoding RNA expression profiles in sub-lethal heat-treated hepatoma carcinoma cells

**DOI:** 10.1186/s12957-017-1194-4

**Published:** 2017-07-21

**Authors:** Qingsong Deng, Shihan Chen, Chunchuan Fu, Jiayun Jiang, Mengda Zou, Yunhua Tan, Xiaofei Wang, Feng Xia, Kai Feng, Kuansheng Ma, Ping Bie

**Affiliations:** Institute of Hepatobiliary Surgery, Southwest Hospital, Third Military Medical University, Chongqing, 400038 China

**Keywords:** Long noncoding RNA, Hepatocellular carcinoma, Sub-lethal heat treatment, Residual cancer, Radiofrequency ablation

## Abstract

**Background:**

Sub-lethal heat treatment characterizes a transition zone of radiofrequency ablation (RFA) which explains hepatocellular carcinoma (HCC) residual cancer occurrence in this area after RFA treatment. The biochemistry of residual cancer cell recurrence is poorly understood, but long noncoding RNAs (lncRNAs) may have aberrant expression that is associated with diverse cancers. Thus, we measured lncRNA gene expression in sub-lethally heat-treated HCC cells using microarray.

**Method:**

Differentially expressed lncRNA and mRNA were measured with an Agilent Human lncRNA + mRNA Array V4.0 (4 × 180 K format) containing 41,000 lncRNAs and 34,000 mRNAs. Bioinformatics analysis was used to assess differentially expressed lncRNA and mRNA. Seven lncRNA and seven mRNA were validated by qRT-PCR analysis in HCC cells.

**Results:**

Genome-wide lncRNA and mRNA expression data in sub-lethal heat-treated SMMC-7721 HCC cells 558 lncRNA and 250 mRNA were significantly up-regulated and 224 lncRNA and 1031 mRNA down-regulated compared to normal cultured SMMC-7721 cells. We demonstrated for the first time that ENST00000570843.1, ENST00000567668.1, ENST00000582249.1, ENST00000450304.1, TCONS_00015544, ENST00000602478.1, TCONS_00001266 and ARC, IL12RB1, HSPA6 were upregulated, whereas STAT3, PRPSAP1, MCU, URB2 were down-regulated in sub-lethally heat-treated HCC cells.

**Conclusions:**

lncRNA expression data in sub-lethally heat-treated HCC cells will provide important insights about lncRNAs’ contribution to HCC recurrence after RFA treatment.

**Electronic supplementary material:**

The online version of this article (doi:10.1186/s12957-017-1194-4) contains supplementary material, which is available to authorized users.

## Background

Liver cancer is the second leading cause of cancer death for men in less developed countries. In more developed countries, it is the sixth leading cause of cancer death among men [1] and hepatocellular carcinoma (HCC) is the most prevalent and malignant type of liver cancer. Radiofrequency ablation (RFA) is commonly used to treat nonresectable and small liver tumors (≤3 cm) and may provide tumor clearance and increase quality of life. However, clinical research results indicate that local recurrence after RFA of liver tumors varies between 2 to 60% [2–4], which is greater than after liver resection. The literature is almost unanimous that local hepatic tumor recurrence after RFA is sooner than after liver resection, why this occurs is not clear.

RFA temperature distribution effectively divides the treatment area into an application, central, transition, and reference zones. For the application and central zones, 60 °C is applied and causes immediate necrosis [5]. Transition zone temperature is 42–60 °C, which is insufficient to kill tumor cells, so some cells survive and can form subsequent tumors. The reference area has no effect on tumor cells.

After RFA, HCC cell biological behavior is modified, and sub-lethal RFA may actually increased malignant transformation of HCC [6] and facilitate rapid progression of residual hepatic VX2 carcinoma [7]. Also, rapid progression of residual tumors via hypoxia inducible factor-1α (HIF-1α)/vascular endothelial growth factor A (VEGFA) pathways can occur and epithelial-mesenchymal transition (EMT) markers have been reported to be expressed at these recurrence sites after RFA treatment [8]. Also, insufficient RFA promotes EMT of HCC cells [9] and malignancy [10]. After insufficient RFA, tumor-associated endothelial cells have enhanced angiogenesis and invasiveness of residual HCC [11], and this may promote tumor angiogenesis via HIF-1α/VEGFA pathway [12]. Incomplete RFA stimulates proliferation of residual renal carcinoma cells [13] and enhances invasiveness and metastasis of residual HCC cancers [14].

Long noncoding RNA (lncRNA) is generally defined as a transcript larger than 200 nucleotides that lacks protein-coding functions. Recently, lncRNAs have been shown to have critical regulatory roles in cancer biology, including genetic imprinting, immune response, tumorigenesis, cellular development, and metabolism [15, 16]. HCC lncRNA expression measured with microarray confirmed that hundreds of lncRNAs were abnormally expressed in HCC tissues [17]. Furthermore, invasion- and metastasis-related lncRNA of HCC have also been identified [18]. However, the role of lncRNA in biological behavioral changes in residual HCC cells in the transition zone of RFA treatment is still unclear.

In the present work, cultured SMMC-7721 cells were subjected to hyperthermia (50 °C) for 10 min to mimic the transition zone produced by RFA, and this is an accepted model at this time [10, 11]. Then, we measured changes in lncRNA expression in HCC cells and estimated the biological significance of these lncRNAs.

## Methods

### Cell culture

Human SMMC-7721, HepG2, and MHCC97-H HCC cells were obtained from the Cell Bank of the Shanghai Institutes for Biological Sciences, Chinese Academy of Sciences (Shanghai, China). Cells were maintained in high-glucose Dulbecco’s modified Eagle’s medium (DMEM) (GIBCO, Invitrogen, Australia) supplemented with 10% fetal bovine serum (FBS) (GIBCO, Invitrogen) and 1% penicillin-streptomycin (GIBCO, Invitrogen) at 37 °C in a 5% CO_2_ atmosphere.

### Sub-lethal heat treatment

SMMC-7721, HepG2, and MHCC97-H cells were sub-lethally heated (50 °C) for 10 min. Heat treatments were carried out by sealing the culture bottle with Parafilm, and submerging the plates in a water bath (HH · W21 · 600S, Shanghai, China) set at 50 °C and returned to the incubation chamber (Series II Water Jacket, Thermo-Scientific, Waltham, MA) for 24 h at 37 °C. Three independent experiments were performed.

### RNA extraction

Total RNA was extracted from sub-lethally heat-treated HCC cells using RNAiso Plus Reagent (TaKaRa, Dalian, China) according to the manufacturer’s instructions. RNA quantification and quality were assured with a NanoDrop ND-2000 spectrophotometer (Thermo-Scientific). RNA integrity and gDNA contamination were confirmed with agarose gel electrophoresis (Additional file 1: Figure S1).

### Microarray analysis

Sample preparation and microarray hybridization and assay were performed by CapitalBio Corporation (Beijing, China). Briefly, RNA was purified with mirVana miRNA Isolation Kit (Ambion, Austin, TX) according to the manufacture’s protocol. cDNA labeled with a fluorescent dye (Cy5 and Cy3-dCTP) was produced using Eberwine’s linear RNA amplification method and subsequent enzymatic reaction as described [19] with improvements using a CapitalBio cRNA Amplification and Labeling Kit (CapitalBio) for greater yields of labeled cDNA. cRNA amplification and labeling is depicted in Additional file 2: Figure S2. lncRNA + mRNA array data were analyzed for summarization, normalization and quality control with GeneSpring software V13.0 (Agilent). To select differentially expressed genes, we used threshold values of ≥2 and ≤ −2-fold changes and a Benjamini-Hochberg corrected *p* value were <0.05 (multiple testing, Benjamini-Hochberg method). Data were Log2-transformed and median centered using genes and the Adjust Data function of CLUSTER 3.0 software then analyzed with hierarchical clustering with average linkages. Finally, we performed tree visualization using Java Treeview (Stanford University School of Medicine, Stanford, CA).

### Construction of the lncRNA-mRNA gene co-expression network

The lncRNA-mRNA co-expression network was constructed based on the correlation between differentially expressed lncRNAs and mRNAs. For each gene pair, a Pearson correlation coefficient was calculated and significant correlation pairs were selected to construct the network. lncRNAs and mRNAs with coefficients >0.99 were selected for network design using the open-source bioinformatics software Cytoscape. In a network analysis, a degree centrality is defined as the links one node has with other nodes. A degree is the simplest and most important measures of a gene centrality within a network and this establishes relative importance [20]. A yellow ellipse represents selected seven up-regulated lncRNAs and a green ellipse represents mRNAs. Red lines represent positive correlations and blue lines represent negative correlations.

### Gene ontology and pathway analysis

KOBAS 2.0 (KEGG Orthology Based Annotation System) was used to perform GO and pathway analysis. Its purpose is to identify significantly enriched pathways and diseases for a set of genes or proteins, using pathway and disease information from multiple databases. In the present study, *p* < 0.05 was a threshold to eliminate pathways related to sub-lethally heat treated HCC cells.

### Liver specific lncRNA analysis

We downloaded liver-specific lncRNA data from deepbase v2.0 (http://rna.sysu.edu.cn/deepBase/heatmap_lncRNA_Table.php?SClade=mammal&SOrganism=hg19&SSource=deepBase2study_humanBody). We screened highly enriched (5.766 to 0.332) and less enriched (−2.98 to 0.445) hepatic lncRNAs according to the expression mean normalization (log2(FPKM)-mean). Then, we identified and compared hepatic lncRNAs with low expression in the liver to hepatic lncRNAs highly expressed in sub-lethally heat-treated HCC cells to determine which lncRNAs were modified.

### Quantitative reverse transcription PCR (qRT-PCR) validation

Total RNA was reverse-transcribed using a PrimeScript RT reagent Kit with gDNA Eraser (Perfect Real Time) (RR047A, TaKaRa, Dalian, China) with a GeneAmp PCR system 2700 (Applied Biosystems, Singapore). Real-time PCR amplification was used to measure lncRNA and mRNA with SYBR Premix Ex Taq II (RR820A, TaKaRa, Dalian, China) with a CFX96 Real-Time System (C1000 Thermal Cycler, Bio-Rad, Singapore) according to the manufacturer’s protocol. Amplification conditions were 95 °C for 30 s, followed by 40 cycles of 95 °C for 5 s and 60 °C for 30 s or 95 °C for 5 s, 55 °C for 30 s and 72 °C for 30 s, with a final extension at 65 °C for 5 s. Primers used are shown in Additional file 3: Tables S1 and Additional file 4: S2. GAPDH was used as a control. lncRNA and mRNA were calculated using the formula 2^−ΔΔCt^, as previously reported [21].

### Statistical analysis

All assays were repeated a minimum of three times. Data were analyzed using SPSS 17.0 software. All quantitative data were expressed as means ± standard deviations (SD). Gene expression in sub-lethally heat-treated HCC cells was compared to non-treated HCC cells. A Student’s t-test was used to compare data between the two groups (*p* < 0.05 was considered a statistically significant difference).

## Results

### Expression profile of lncRNA and mRNA in sub-lethally heat-treated HCC cells

To identify lncRNA and mRNA modified by sub-lethal heat treatment in HCC, we applied RFA (50 °C) to SMMC-7721 HCC cells continuously for 10 min to simulate the RFA transition zone.

**Fig. 1:**
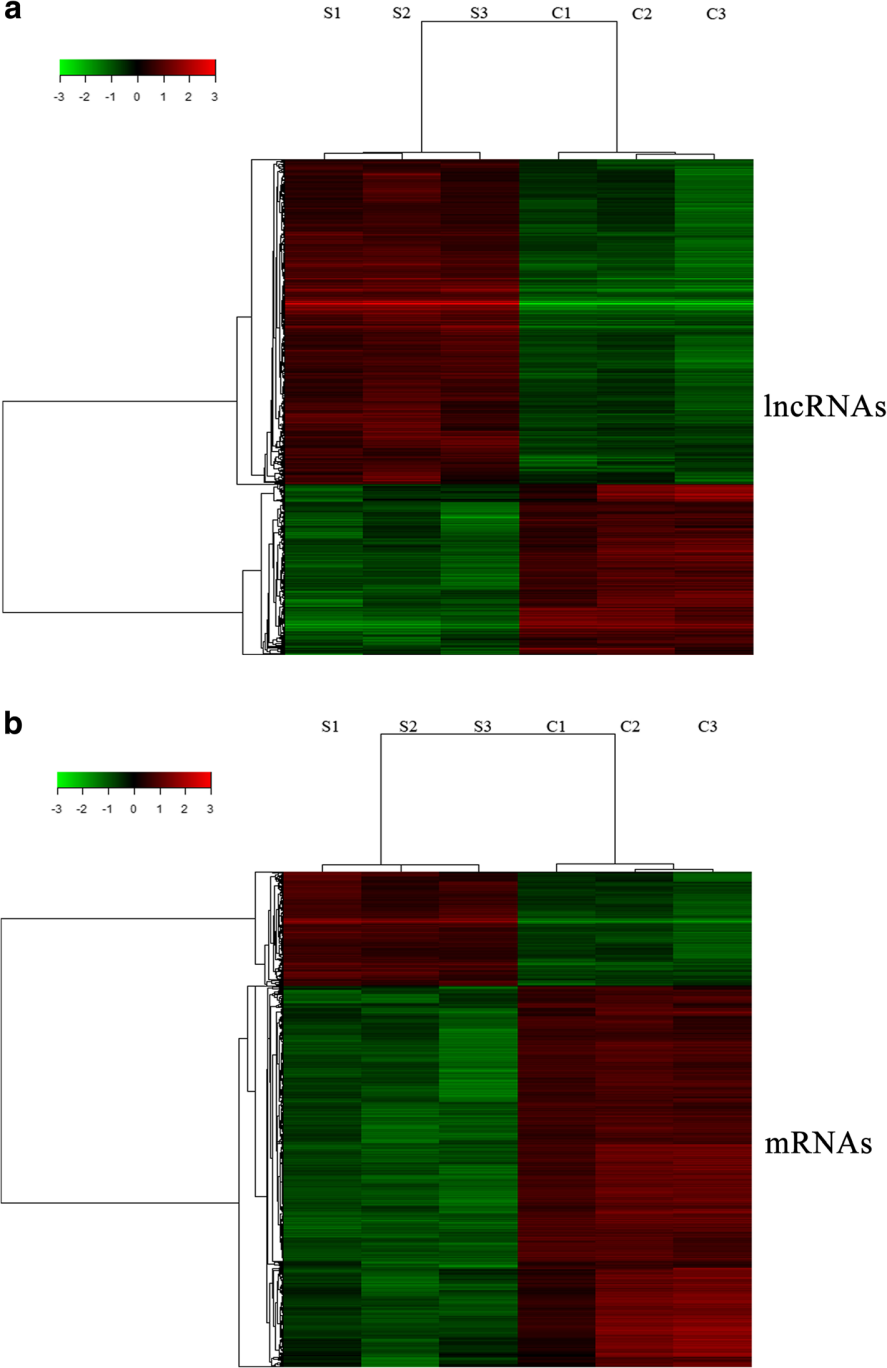
Heat maps of differential expression and hierarchical clustering of lncRNAs (**a**) and mRNAs (**b**) in sub-lethally heat-treated HCC cells and untreated HCC cells. *S* sub-lethal heat treatment; *C* Control (Normal culture)

Fold-changes (treated vs non-treated HCC cells) and *p (Corr)*-values were calculated from normalized expression data. Using microarray analysis (Fig. 1), we identified 782 lncRNAs and 1,281 mRNAs to be significantly differentially expressed in sub-lethally heat-treated HCC cells (foldchange >2.0, *p (Corr) <* 0.05). Among these, 558 lncRNA and 250 mRNA were consistently upregulated in sub-lethally heat-treated HCC cells, and 224 lncRNA and 1,031 mRNA were consistently downregulated (Additional file 5: Table S3). lncRNA XR_428946.1 expression (fold-change = 227.8) was significantly upregulated, but expression of XR_243720.2 (foldchange = 15.3) was dramatically downregulated. These lncRNA may play important roles in the occurrence and development of residual hepatocellular carcinoma cells after insufficient RFA treatment. Activity-regulated cytoskeleton-associated protein (ARC) expression (fold-change = 130.2) was significantly upregulated, and expression of chemokine (C-X-C motif) ligand 1 (CXCL1) (foldchange = 26.0) was dramatically downregulated.

**Fig. 2:**
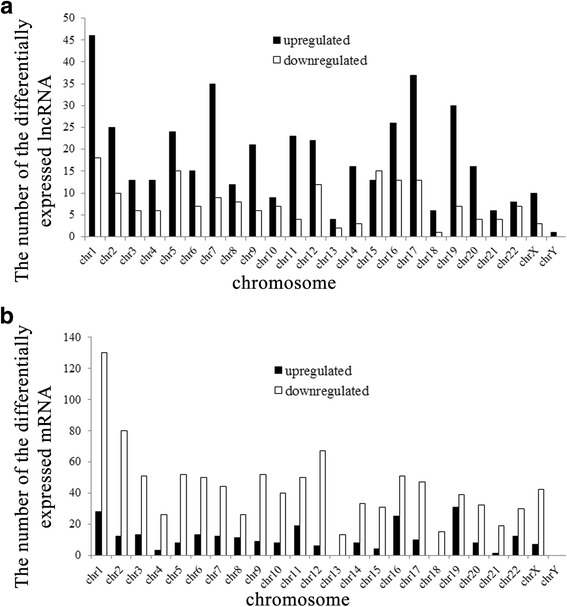
Up- and down-regulated lncRNA (**a**) and mRNA (**b**) on each chromosome in comparison between sub-lethally heat-treated HCC cells and untreated HCC cells

Differentially expressed lncRNAs and mRNAs in sub-lethally heat-treated HCC cells were not distributed equally on all chromosomes. Distribution pattern analysis of differentially expressed lncRNAs and mRNAs on chromosomes confirmed that chromosome 1 (chr1) had the most differentially expressed lncRNAs and mRNAs, chromosome Y (chrY) had the least differentially expressed lncRNAs and mRNAs (Fig. 2)

.

**Fig. 3:**
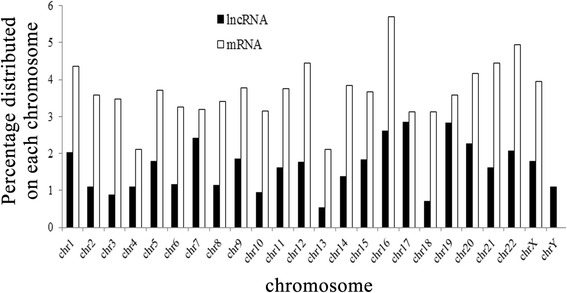
Distribution of significantly differentially expressed lncRNAs and mRNAs on each chromosome in comparisons between sub-lethally heat-treated HCC cells and untreated HCC cells

We found that chromosome 1 has the most differentially expressed lncRNAs and mRNAs, but due to many genes on chromosome 1, the proportion of differentially expressed genes was not the greatest. The most differentially expressed lncRNA were on chromosome 17, whereas mRNA was on chromosome 16. Chromosome 16 has the greatest fraction of differentially expressed lncRNA and mRNA (Fig. 3).

### lncRNA classification and subgroup analysis

Analysis of the genomic context of lncRNAs that were changed in sub-lethally heat-treated HCC cells may predict or identify their functional role. Therefore, we analyzed associations between lncRNAs and mRNAs to identify putative functional relationships, categorizing these as sense, antisense, intronic, divergent or intergenic types. The most differentially expressed lncRNA are intergenic types of lncRNA. Data for these relationships appear in Tables 1 and Additional file 6: S4 and Additional file 7: S5.

**Table 1:** Overview of the subgroups of differentially expressed lncRNAs in sub-lethally heat-treated HCC cells

Expression	Sense lncRNAs	Antisense lncRNAs	Intronic lncRNAs	Divergent lncRNAs	Intergenic lncRNAs
Upregulated Downregulated	6 4	154 81	12 10	34 10	188 59

### Liver specific lncRNA analysis

We found 560 liver-specific highly expressed and 400 under expressed lncRNAs in the deepbase, and compared it with microarray data, and 3 lncRNA had modified expression after sub-lethal heat treatment. Also, two lncRNA(GAS5 and PVT1) were up-regulated and one was (OIP5-AS1) downregulated. These data appear in Additional file 8: Table S6. These three lncRNA may have a role in the biochemistry of residual cancer cell recurrence after RFA treatment.

### qRT-PCR analysis of lncRNA and mRNA expression

**Fig. 4:**
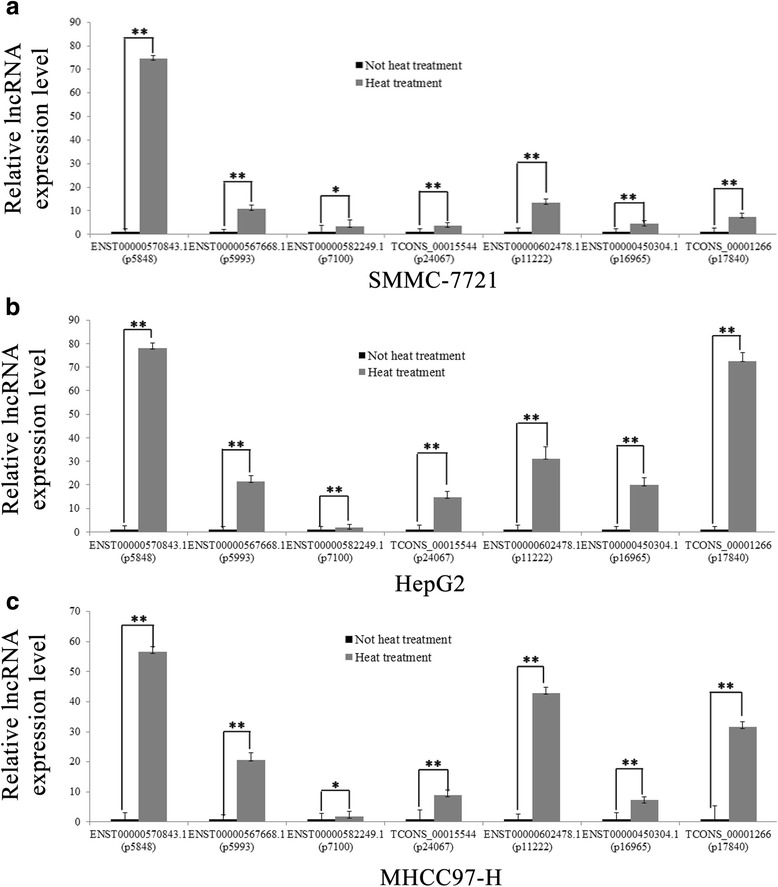
Validation of five differentially expressed lncRNAs and six mRNAs in SMMC-7721, HepG2 and MHCC97H cell lines using qRT-PCR. Relative expression of lncRNA and mRNA in untreated HCC cells was calculated by dividing the expression of corresponding lncRNAs and mRNA in sub-lethally heat-treated HCC cells. Relative expression greater than 1 indicates upregulation; relative expression less than 1 indicates downregulation

**Fig. 5:**
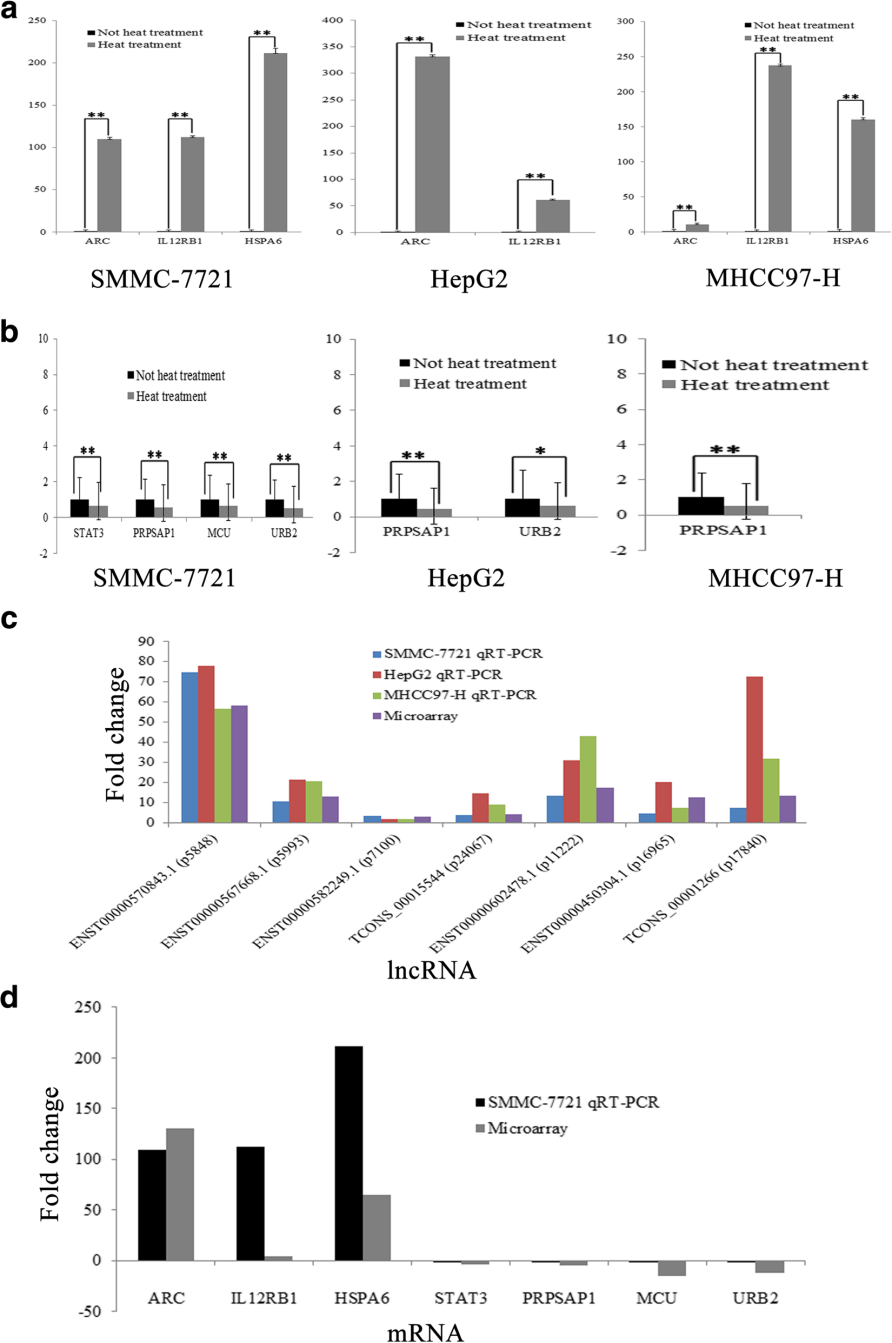
Co-expression network of five upregulated lncRNA from comparisons between sub-lethally heat-treated HCC cells and untreated HCC cells, with differentially expressed mRNA. Correlation >0.99 or <0.99 and *p <* 0.05 were recognized as co-expression. *Yellow ellipse* represents five up-regulated lncRNAs; *green ellipse* represents mRNA. *Red lines*: positive correlation, *blue lines*: negative correlation. *Node size* indicates node degrees (number of neighbors)

According to fold-differences, co-expression, and liver-specific lncRNA analysis, we initially identified many candidate lncRNA and mRNA for validation. Seven lncRNAs (ENST00000570843.1, ENST00000567668.1, ENST00000582249.1, ENST00000450304.1, TCONS_00015544, ENST00000602478.1, TCONS_00001266 and seven mRNAs (ARC, IL12RB1, HSPA6, STAT3, PRPSAP1, MCU, URB2) were examined with qRT-PCR (Fig. 4, Fig. 5a, b) and Tables 2 and 3 depict data regarding their characteristics. lncRNA qRT-PCR and microarray data were consistent (Fig. 5c, d), but mRNA expression changes in HepG2 and MHCC97-H cell lines were not consistent with that in SMMC-7721 cells. For example, HSPA6 gene expression in HepG2 cells was not statistically significantly different between the heat treatment and no treatment groups. (Fig. 5a, b)

**Table 2:** Characteristics of qRT-PCR lncRNAs

lncRNA	Expression	Chromosome	Strand	Start	End	Class
ENST00000570843.1(p5848)	Up	16	−	3206736	3207484	Intergenic
ENST00000567668.1(p5993)	Up	16	−	33950098	33957115	Intergenic
ENST00000582249.1(p7100)	Up	17	−	80434472	80435800	Antisense
TCONS_00015544(p24067)	Up	8	−	144795187	144796371	Intergenic
ENST00000602478.1(p11222)	Up	22	+	43011249	43011913	Intergenic
ENST00000450304.1(p16965)	Up	9	+	139443581	139444345	Antisense
TCONS_00001266(p17840)	Up	1	+	212731195	212734165	Intergenic

**Table 3:** Characteristics of qRT-PCR mRNAs

mRNA	Expression	GenBank Accession	Gene name	Genomic coordinates	Cytoband
ARC	Up	NM_015193	Activity-regulated cytoskeleton-associated protein	chr8:143692638-143692579	hs|8q24.3
IL12RB1	Up	NM_153701	Interleukin 12 receptor, beta 1	chr19:18183032-18182973	hs|19p13.11
HSPA6	Up	NM_002155	Heat shock 70 kDa protein 6 (HSP70B’)	chr1:161495332-161495391	hs|1q23.3
STAT3	Down	NM_213662	Signal transducer and activator of transcription 3 (acute-phase response factor)	chr17:40474463-40474404	hs|17q21.2
PRPSAP1	Down	NM_002766	Phosphoribosyl pyrophosphate synthetase-associated protein 1	chr17:74307297-74307238	hs|17q25.1
MCU	Down	NM_138357	Mitochondrial calcium uniporter	chr10:74646758-74646817	hs|10q22.1
URB2	Down	NM_014777	URB2 ribosome biogenesis 2 homolog (S. cerevisiae)	chr1:229795276-229795335	hs|1q42.13

### Construction of the lncRNA-mRNA co-expression network

**Fig. 6:**
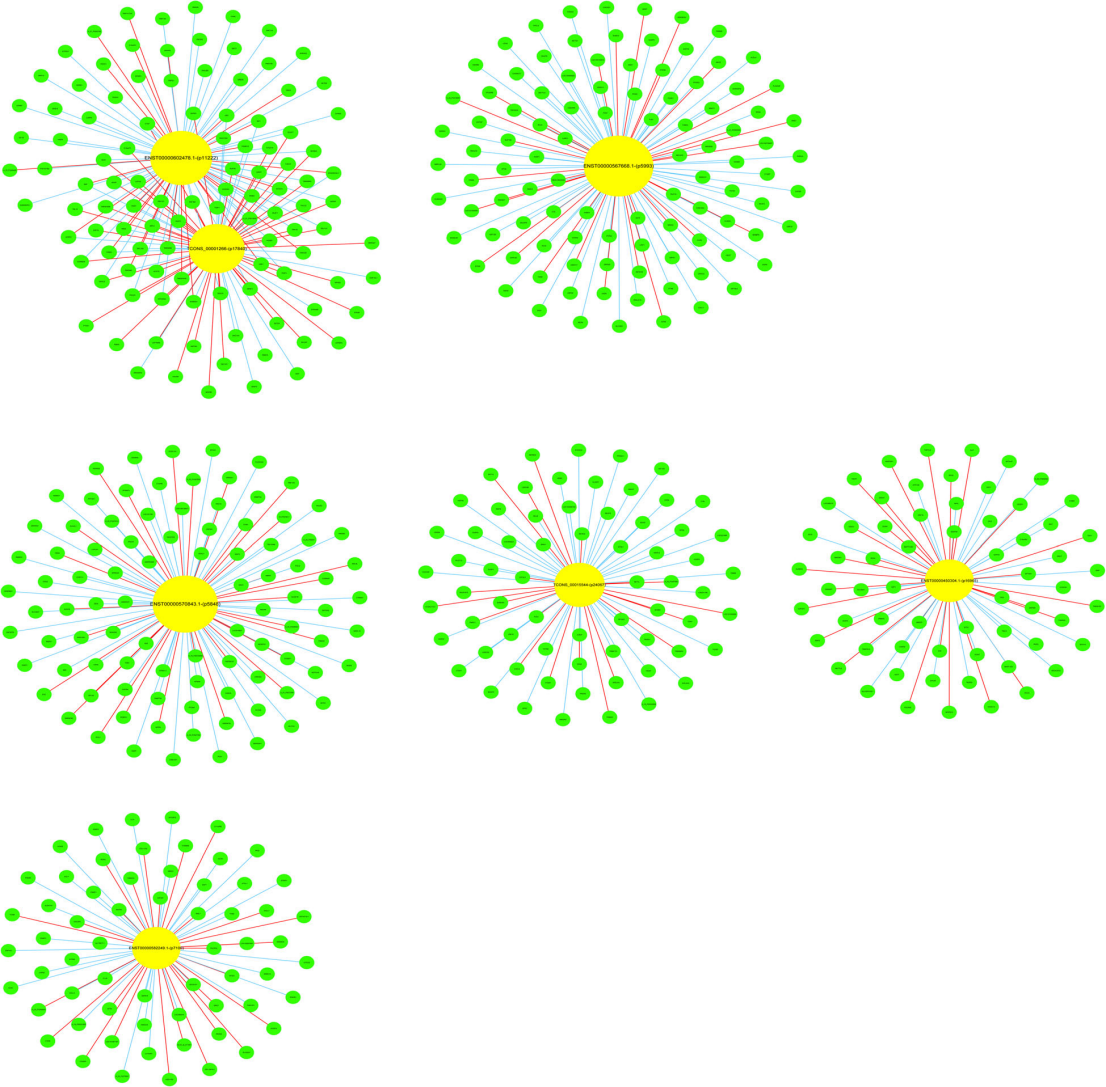
Gene ontology (GO) enrichment analysis for differentially regulated mRNA. GO analysis of mRNA according to biological processes (**a**), molecular function (**b**) and cellular component (**c**)

To understand the correlation between differentially expressed lncRNAs and mRNAs in sub-lethally heat-treated HCC cells, we constructed a lncRNA-mRNA co-expression network but the data were too numerous, so we selected seven lncRNAs and used qRT-PCR validation to create a network diagram (Fig. 6, Additional file 9: Table S7). We identified seven pairs of co-expressed lncRNAs and mRNAs composed of 548 mRNA (37.61% [548/1457]) of all differentially expressed mRNAs) and 219 pairs were positively correlated (15.03% of all correlations). This network indicated that one lncRNA could correlate with one or tens of mRNA and different lncRNA correlated with the same mRNA. Moreover, this network could be used to predict target genes of these lncRNAs.

Five of seven qRT-PCR validated lncRNA could predict target mRNA. STAT3 is the target gene of lncRNA ENST00000570843.1 and STAT3 was downregulated according to microarray and qRT-PCR analysis. Many target mRNA of these lncRNA were downregulated (Additional file 10: Table S8).

### GO and pathway analyses

**Fig. 7:**
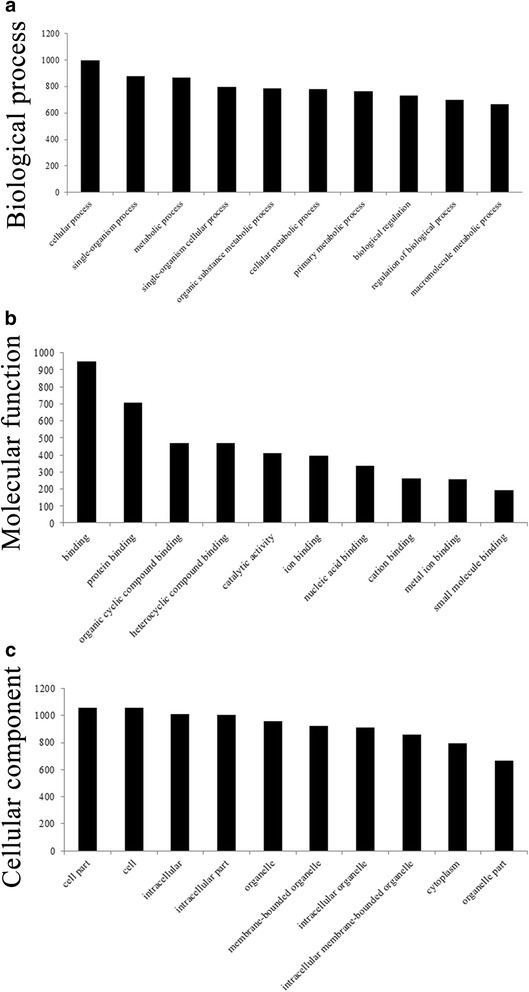
GO enrichment analysis for target genes of differentially regulated lncRNAs. GO analysis of mRNA according to biological processes (**a**), molecular function (**b**) and cellular component (**c**)

GO analysis was performed to identify genes and measure gene product enrichment involved in biological processes, cellular components and molecular functions. We noted that significantly different transcripts between sub-lethally heat-treated HCC cells and untreated HCC cells were mainly associated with cellular process (ontology: biological process), binding (ontology: molecular function), and the cell component (ontology: cellular component) (Fig. 7, Additional file 11: Table S9). Pathway analysis indicated that 265 pathways corresponded to significantly different transcripts and that the most enriched network was “metabolic pathways,” which was comprised of 108 targeted genes (Additional file 12: Table S10).

**Fig. 8:**
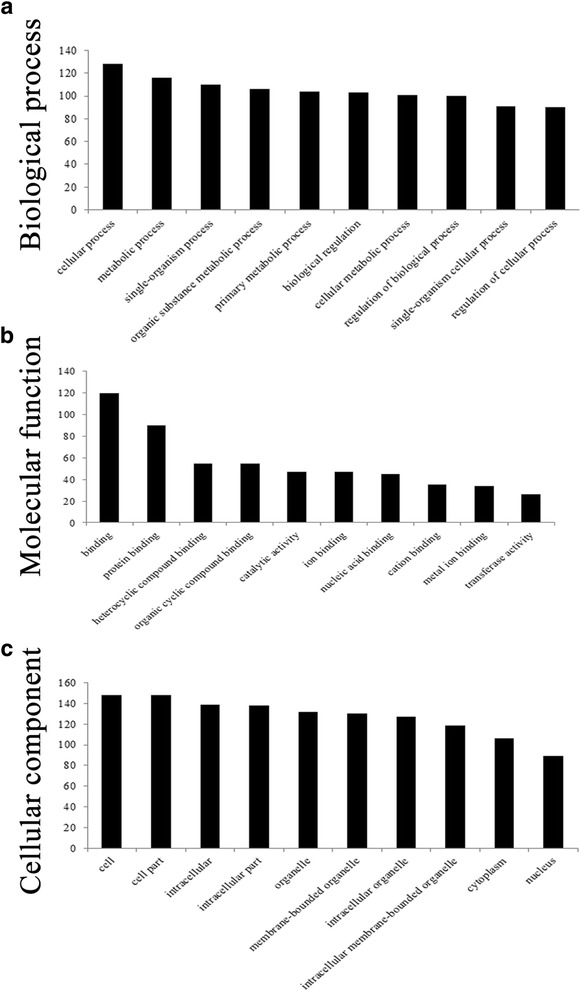
GO enrichment analysis of target genes of differentially regulated lncRNAs. GO analysis of mRNA according to biological processes (**a**), molecular function (**b**) and cellular component (**c**)

Target genes of significantly different lncRNAs assessed with GO indicated that these target genes were mainly associated with cellular process (ontology: biological process), binding (ontology: molecular function) and the cell (ontology: cellular component) (Fig. 8, Additional file 13: Table S11). Pathway analysis indicated that 142 pathways were associated with significantly different lncRNAs and again the most enriched network was “metabolic pathways,” which was comprised of nine targeted genes (Additional file 14: Tables S12).

## Discussion

RFA is commonly used for treating HCC and this usually produces a transition zone in which the temperature does not achieve therapeutic efficacy, so it is sub-lethal to these cells. These residual tumor cells can return and rapidly produce subsequent tumors, representing a significant drawback for RFA. Recently, research suggests that lncRNAs are important in complicated diseases such as cancer [22]. Several lncRNA have been identified as being involved in the development and progression of HCC, such as lncRNA-HEIH [23], HOTAIR [24, 25], lncRNA MVIH [26], lncRNA-Dreh [27], HOTTIP/HOXA13 [28, 29], URHC [30], PCNA-AS1 [31], UFC1 [32], HULC [33], CCAT1 [34], PVT1 [35], ANRIL [36, 37], ZEB1-AS1 [38], PANDAR [39], DANCR [40]. Some of these lncRNA were also identified in our microarray data. For example, PVT1 and DANCR were upregulated in sub-lethally heat-treated HCC cells. Research indicates that BC017743, ENST00000395084, NR_026591, NR_015378, and NR_024284 were up-regulated, whereas NR_027151, AK056988, and uc003yqb.1 were down-regulated in HCC samples and adjacent non-tumor tissues according to microarray analysis [17]. The relationship between lncRNAs and HCC cell invasion and metastasis was identified using the same method [18]. Long noncoding RNA expression in TGF-β2-induced epithelial-mesenchymal transition has been identified [41] and investigators have reviewed the important role for lncRNA in hepatitis B virus-induced liver cancer [42], but few studies exist to describe changes in lncRNA in sub-lethally heat-treated HCC cells that recover after treatment.

Here, we report that 782 lncRNA and 1281 mRNA were significantly differentially expressed compared to untreated SMMC-7721 cells. Bioinformatic analyses confirmed that differentially expressed lncRNAs and mRNAs using GO, as well as pathway and co-expression network analysis had value for target and transcript factor prediction. Subsequent analysis of hepatic-specific lncRNAs and validation of microarray results by qRT-PCR were also performed. Data from GO and pathway analysis indicated that differentially expressed mRNAs and lncRNAs are related to metabolic pathways, indicating that metabolic pathways in sub-lethally heat-treated HCC cells undergo energy changes that allow adaptation and survival. We uploaded these data to the GEO web site (accession # GSE99351).

We selected seven lncRNA and seven mRNA for qRT-PCR analysis and these data are consistent with microarray data. But the mRNA expression is not the same in the three HCC cells, probably because different cell lines have different biological behaviors and expression profiles. Then, we constructed lncRNA-mRNA co-expression networks for seven lncRNAs. ENST00000570843.1 expression was the most changed, so in the future, we will study its role in proliferation and recurrence of residual cancer cells after RFA treatment.

We previously reported that RFA combined with sorafenib can prolong patient survival [43]. However, chemo-resistance is common and influences patient prognosis. Here, we report [44] data for lncRNA expression in chemo-resistant HCC cells in which we identified 120 differentially expressed lncRNAs, of which 61 were up-regulated and 59 were down-regulated. The underlying pathways of these differences in expression were related to cell death, proliferation, and cellular response to stimuli, and these included the p53, ErbB, and MAPK pathways. There is an interest in using lncRNAs as biomarkers of cancer as some investigators report that lncRNA expression data obtained with microarray from plasma of HCC and chronic hepatitis B patients had markers that may help diagnose HCC. Specifically, they identified lncRNA-urothelial carcinoma associated-1 (lncRNA-UCA1) and WD repeat containing antisense to TP53 (WRAP53) as novel biomarkers for noninvasive diagnosis of HCC [45]. Serum expression of uc001ncr and AX800134 may also be a biomarker for diagnosing HCC, especially for patients with AFP <400 ng/ml or with early-stage disease (BCLC 0 + A) [46]. Thus, lncRNA may be novel therapeutic targets for cancer [47].

Our choice of 50 °C heat applied for 10 min was selected based on observations that this best simulated the RFA transition zone when treating HCC. Also, tumor cells did not die after this heat application but their proliferation and invasiveness was enhanced after culture. This may cause more rapid tumor recurrence compared to surgical resection. The timing of 10 min best approximates clinical RFA applications. Finally, this heat applied at this time did not kill all cancer cells, but they recovered after 3 weeks (data not shown). After application of 47 °C for 10 min, cancer cells did not have altered morphology and did not change significantly after a lengthy culture. Therefore, our temperature and duration of application may best replicate heat stress experienced by residual cancer cells in the RFA transition zone.

Study limitations include sub-lethal RFA and lncRNA expression measurements after a short interval. lncRNA expression changes may not occur this rapidly in clinical specimens. In addition, we could not obtain HCC tissue specimens during or after RFA treatment because HCC biopsies are controversial in our institution (rarely produced). Therefore, HCC tissue specimens were not used for microarray analysis. So, we will quantify ENST00000570843.1 in blood samples (see above). In summary, we described global expression profiles of lncRNAs and mRNAs in sub-lethally heat-treated HCC cells using lncRNA and mRNA microarrays. With bioinformatics predictions, we identified target genes that are correlated with differentiation of seven candidate lncRNAs. Further studies of functional analysis of these lncRNA are needed to provide more conclusive evidence about their regulatory mechanisms. Collectively, our results suggest that lncRNAs are important regulators of transition zone tumor recurrence and may guide further investigation into mechanisms of lncRNAs that regulate residual cancer cell return after RFA treatment.

## Conclusions

In this study, we simulated the transition zone (area of residual cancer occurrence) of RFA with sub-lethal heat treatment of the cultured cells. We obtained long noncoding RNA expression profiles in sub-lethal heat-treated hepatoma carcinoma cells. Our results provide theoretical support for residual cancer recurrence in the transition zone of RFA treatment. We provided new ideas for biological change of residual cancer cells.

## Additional files

Additional file 1: Figure S1. RNA quality. ① and ⑧: DL2000 Marker; ②: Normal cultured HCC cells(The first repeat experiment); ③: sub-lethally heat-treated HCC cells(The first repeat experiment); ④Normal cultured HCC cells(The second repeat experiment); ⑤sub-lethally heat-treated HCC cells(The second repeat experiment); ⑥Normal cultured HCC cells(The third repeat experiment); ⑦ sub-lethally heat-treated HCC cells(The third repeat experiment); (TIF 1244 kb)

Additional file 2: Figure S2. cRNA amplification and labeling procedures (TIF 251 kb)

Additional file 3: Table S1. lncRNA qRT-PCR primers and product size. (DOCX 18 kb)

Additional file 4: Table S2. mRNA qRT-PCR primers and product size. (DOCX 17 kb)

Additional file 5: Table S3. Significantly differentially expressed lncRNAs and mRNAs. (XLS 2545 kb)

Additional file 6: Table S4. Classification and subgroup analysis of upregulated lncRNAs. (XLSX 206 kb)

Additional file 7: Table S5. Classification and subgroup analysis of downregulated lncRNAs. (XLSX 92 kb)

Additional file 8: Table S6. Liver specific lncRNA analysis. (XLSX 349 kb)

Additional file 9: Table S7. Significant correlation of cytoscape network. (XLS 51 kb)

Additional file 10: Table S8. Target prediction summary. (XLS 2 kb)

Additional file 11: Table S9. N vs C GO enrichment. (XLSX 1980 kb)

Additional file 12: Table S10. N vs C pathway enrichment. (XLSX 351 kb)

Additional file 13: Table S11. Target GO enrichment. (XLSX 443 kb)

Additional file 14: Table S12. Target pathway enrichment. (XLSX 107 kb)

## Abbreviations

EMT: Epithelial-mesenchymal transition
HCC: Hepatocellular carcinoma
HIF-1α: Hypoxia inducible factor-1α
lncRNAs: Long noncoding RNAs
RFA: Radiofrequency ablation
VEGFA: Vascular endothelial growth factor A
