# Supplementary figures and images for: Long noncoding RNA expression profiles in sub-lethal heat-treated hepatoma carcinoma cells
Source: World J Surg Oncol. 2017 Jul 21;15:136. doi: 10.1186/s12957-017-1194-4 (PMC5521104; doi:10.1186/s12957-017-1194-4)

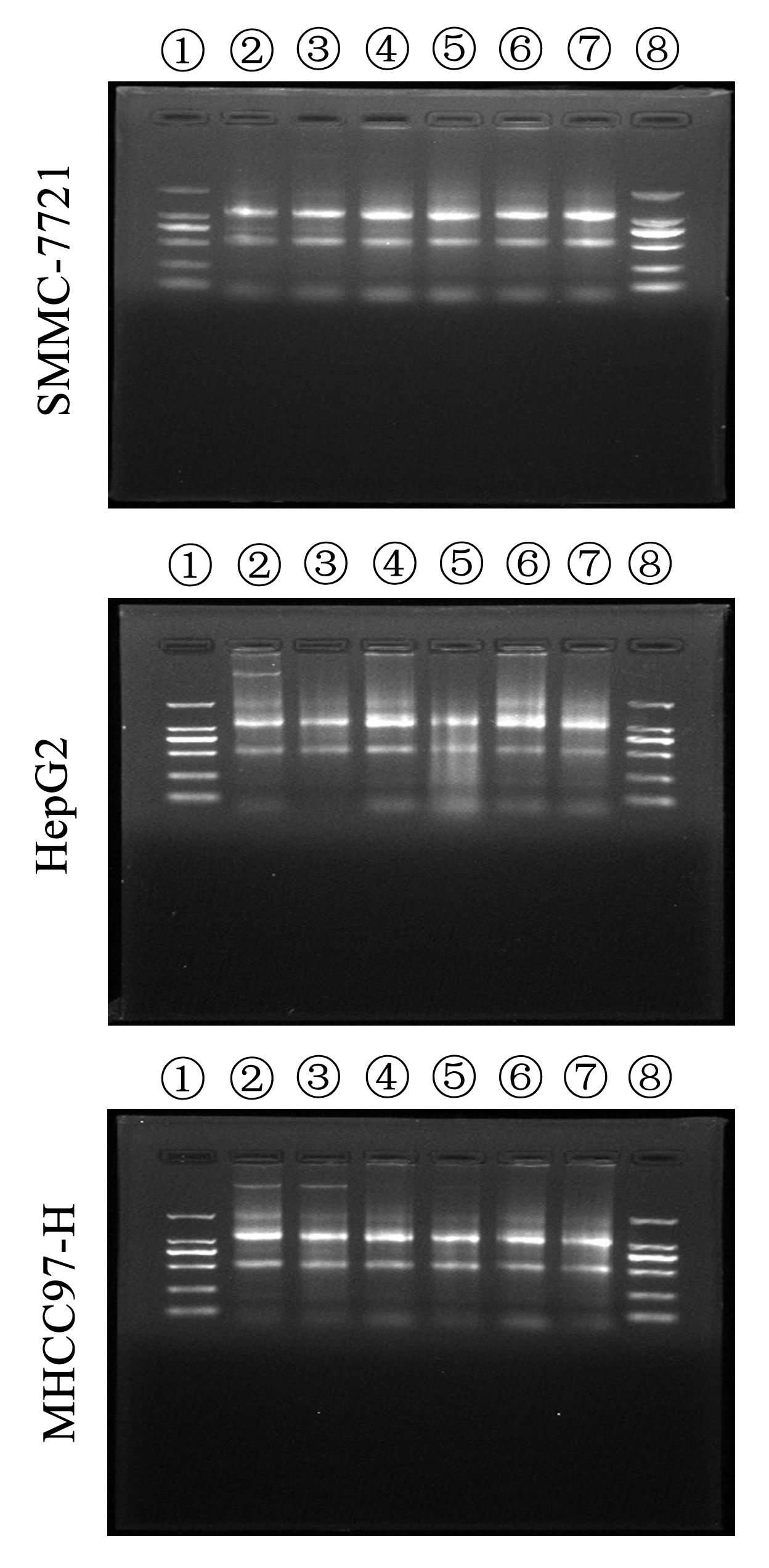

Supplement: Supplementary file 1 — RNA quality. ① and ⑧: DL2000 Marker; ②: Normal cultured HCC cells(The first repeat experiment); ③: sub-lethally heat-treated HCC cells(The first repeat experiment); ④Normal cultured HCC cells(The second repeat experiment); ⑤sub-lethally heat-treated HCC cells(The second repeat experiment); ⑥Normal cultured HCC cells(The third repeat experiment); ⑦ sub-lethally heat-treated HCC cells(The third repeat experiment); (TIF 1244 kb) [file 12957_2017_1194_MOESM1_ESM.tif]

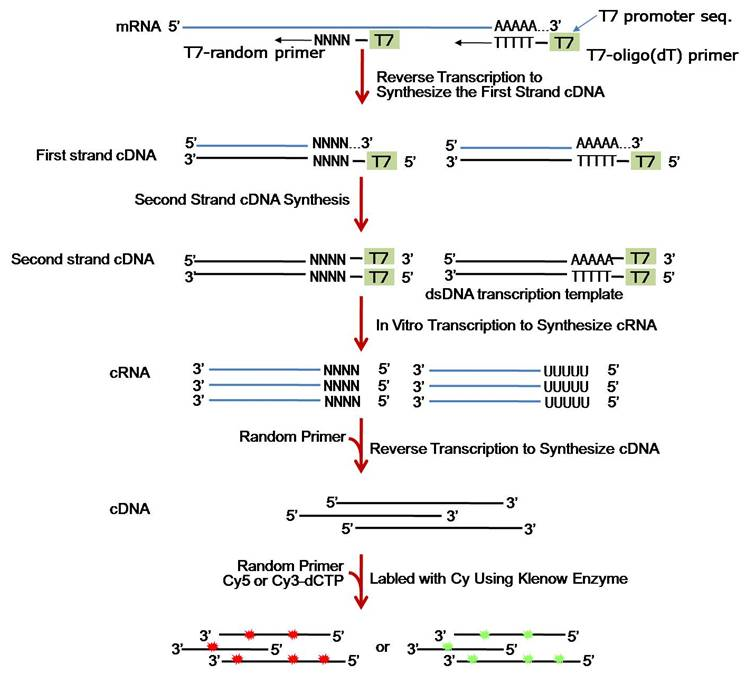

Supplement: Supplementary file 2 — cRNA amplification and labeling procedures (TIF 251 kb) [file 12957_2017_1194_MOESM2_ESM.tif]
