# Supplementary material for: Long noncoding RNA expression profiles in sub-lethal heat-treated hepatoma carcinoma cells
Source: World J Surg Oncol. 2017 Jul 21;15:136. doi: 10.1186/s12957-017-1194-4 (PMC5521104; doi:10.1186/s12957-017-1194-4)
Supplement: Supplementary file 3 — lncRNA qRT-PCR primers and product size. (DOCX 18 kb) [file 12957_2017_1194_MOESM3_ESM.docx]

**Table S1** lncRNA qRT-PCR primers and product size.

| *lncRNA ID* | *Forward primer sequence* | | *Reverse primer sequence* | *Size of PCR Product (bp)* | |
| --- | --- | --- | --- | --- | --- |
| ENST00000570843.1(p5848)  ENST00000582249.1(p7100)  TCONS_00015544(p24067)  ENST00000567668.1(p5993)  ENST00000602478.1(p11222)  ENST00000450304.1(p16965)  TCONS_00001266(p17840)  GAPDH | | 5′-ATCTCAGGGTCGTGGGTTC-3′  5′-CCCACAGAGACACAGAAGCA-3′  5′-CTTCACACACACCCAGCATT-3′  5′-GTGTGGTGGAGAAAGGGAAG-3′  5′-CCGACCTTATTCACGCCTAA-3′  5′-CCATCTGCCTTTCTCCACTT-3′  5′-GTGATGAAGGTGGGCTGTG-3′  5′-CAGGAGGCATTGCTGATGAT-3′ | 5′-TTGGTTGGTGGAGCGTTT-3′  5′-TCTTGAAATCCTGCCCTCA-3′  5′-ACAACAGCACACGGATGGT-3′  5′-GTGCCCAGGTATGGAAATGT-3′  5′-ACAGCCAGCGACAAGACTG-3′  5′-CCGAATCCATCCTAGAGCAA-3′  5′-CTGCTGAAAGGCACTGAGATT-3′  5′-GAAGGCTGGGGCTCATTT-3′ | | 170  126  127  120  145  157  101  138 |
