# Supplementary material for: Long noncoding RNA expression profiles in sub-lethal heat-treated hepatoma carcinoma cells
Source: World J Surg Oncol. 2017 Jul 21;15:136. doi: 10.1186/s12957-017-1194-4 (PMC5521104; doi:10.1186/s12957-017-1194-4)
Supplement: Supplementary file 4 — mRNA qRT-PCR primers and product size. (DOCX 17 kb) [file 12957_2017_1194_MOESM4_ESM.docx]

**Table S2** **mRNA qRT-PCR primers and product size.**

| *GeneSymbol* | *Forward primer sequence* | *Reverse primer sequence* | *Size of PCR Product (bp)* |
| --- | --- | --- | --- |
| ARC  IL12RB1  HSPA6  STAT3  PRPSAP1  MCU  URB2  GAPDH | 5′-GCTCCGTGAAGAACTGGGTG-3′  5′-CCTGGCACGGAGGTCACTTA-3′  5′-GCAAGACAAGTGTCGGGAAGT-3′  5′-GGAAGAGGCGGCAACAGATT-3′  5′-CACTCCCCGCCTATGGTCA-3′  5′-TGTTGTGCCCTCTGATGATGTT-3′  5′-GGCTGCTACTGAACTGCCCACT-3′  5′-CTTTGGTATCGTGGAAGGACTC-3′ | 5′-GTCTGGTACAGGTCCCGCTT-3′  5′-ACCCGAGAGATAGGGCATCTT-3′  5′-CCCCATAGAGCCTGGAGAAGA-3′  5′-TGTTGACGGGTCTGAAGTTGAG-3′  5′-TCCACGATGATTGCGATGC-3′  5′-CAACTCTGTCAATTCCCCGATC-3′  5′-CATCTAAGACAGGCCCGACCA-3′  5′-GTAGAGGCAGGGATGATGTTCT-3′ | 157  108  128  112  128  173  150  132 |
